# Oxidative Stress and Regulation of *Pink1* in Zebrafish (*Danio rerio*)

**DOI:** 10.1371/journal.pone.0081851

**Published:** 2013-11-26

**Authors:** Madhusmita Priyadarshini, Lori A. Orosco, Pertti J. Panula

**Affiliations:** 1 Neuroscience Center and Institute of Biomedicine/Anatomy, University of Helsinki, Helsinki, Finland; 2 Carnegie Institution for Science, Department of Embryology, Baltimore, Maryland, United States of America; University of Sheffield - MRC Centre for Developmental and Biomedical Genetics, United Kingdom

## Abstract

Oxidative stress-mediated neuronal dysfunction is characteristic of several neurodegenerative disorders, including Parkinson’s disease (PD). The enzyme tyrosine hydroxylase (TH) catalyzes the formation of L-DOPA, the rate-limiting step in the biosynthesis of dopamine. A lack of dopamine in the striatum is the most characteristic feature of PD, and the cause of the most dominant symptoms. Loss of function mutations in the PTEN-induced putative kinase (PINK1) gene cause autosomal recessive PD. This study explored the basic mechanisms underlying the involvement of *pink1* in oxidative stress-mediated PD pathology using zebrafish as a tool. We generated a transgenic line, *Tg*(*pink1:EGFP*), and used it to study the effect of oxidative stress (exposure to H_2_O_2_) on *pink1* expression. GFP expression was enhanced throughout the brain of zebrafish larvae subjected to oxidative stress. In addition to a widespread increase in *pink1* mRNA expression, mild oxidative stress induced a clear decline in tyrosine hydroxylase 2 (*th2*), but not tyrosine hydroxylase 1 (*th1*) expression, in the brain of wild-type larvae. The drug L-Glutathione Reduced (LGR) has been associated with anti-oxidative and possible neuroprotective properties. Administration of LGR normalized the increased fluorescence intensity indicating *pink1* transgene expression and endogenous *pink1* mRNA expression in larvae subjected to oxidative stress by H_2_O_2_. In the *pink1* morpholino oliogonucleotide-injected larvae, the reduction in the expression of *th1* and *th2* was partially rescued by LGR. The *pink1* gene is a sensitive marker of oxidative stress in zebrafish, and LGR effectively normalizes the consequences of mild oxidative stress, suggesting that the neuroprotective effects of *pink1* and LGR may be significant and useful in drug development.

## Introduction

Parkinson’s disease (PD), a common neurodegenerative disorder, is characterized by the degeneration of nigrostriatal dopamine (DA) neurons, a reduction in striatal DA levels and a decreased DA biosynthetic capacity [1]. Oxidative stress has been implicated as a factor mediating the initiation and progression of many neurodegenerative diseases, including PD. Monogenic variants have been found to be responsible for nearly 10% of the cases in the familial form of PD [2]. Identification of the familial genes responsible for PD has revealed novel proteins and pathways that are likely to be relevant in the pathogenesis of the disease. Current evidence suggests that mitochondrial insufficiency and oxidative stress together act as central players in the pathogenesis of both sporadic and genetic forms of the disease [3-6].

One of the familial genes, PINK1 (PTEN-induced putative kinase 1), is responsible for PARK6-associated autosomal recessive PD (ARPD) [7]. It is the second most common gene after *parkin* to be responsible for the early onset form of the disease, and shows considerable variation across different ethnic groups [8]. PINK1 has been reported to show ubiquitous expression, but higher expression has been found in the brain, myocardium of the heart and testes [9,10]. Given its subcellular localization in the mitochondria and also the cytosol, it is not surprising that PINK1 plays a role in the normal biology of mitochondria, including fission and fusion mechanisms [11]. PINK1 is essential for proper mitochondrial function and is neuroprotective against intrinsic and extrinsic physiological cellular stress. The loss of PINK1 in mice is associated with reduced activities of complex I, II, and aconitase, which are highly sensitive to oxidative stress [12]. Our previous study on the zebrafish model of PINK1 dysfunction identified HIF signaling as one of several significant pathways to be affected [13]. This reinforced the hypothesis that oxidative stress and/or hypoxic stress as a consequence of the loss of PINK1 may lead to PD. It is known that glutathione in the reduced form acts as an antioxidant [14]. The drug L-Glutathione Reduced (LGR) has previously been used to rescue some of the defects caused by PINK1 deficiency [13], but no comprehensive studies using this model have been carried out. Increased oxidative stress is the primary mechanism by which environmental toxins have been associated as potential risk factors for PD [15]. Tyrosine hydroxylase (TH) catalyzes the rate-limiting step in the biosynthesis of dopamine and other catecholamines. Differences have been noted in the concentration and availability of this enzyme and its cofactors in disease states such as PD [1]. In PINK1-deficient larval zebrafish, tyrosine hydroxylase 1 and 2 (*th1* and *th2*) are both significantly reduced [16].

Taking into account the various genetic tools available to genetically manipulate the zebrafish genome, we sought to identify and characterize the zebrafish PINK1 promoter locus to drive GFP expression using the Tol2 transposition method. The Tol2 transposon vector has a potential to carry larger inserts without reducing its transpositional activity, allowing for highly effective stable integration of exogenous DNA [17]. How different organisms handle oxidative stress *in vivo* is poorly understood. Therefore, by using the wild-type strain, larvae with *pink1* deficiency produced by morpholino oligonucleotides, and the PINK1 promoter-driven transgenic fish line, we studied the effect of oxidative stress and evaluated the therapeutic potential of LGR in this model. 

## Materials and Methods

### Fish maintenance

Zebrafish of the Turku line, used for more than a decade in experimental work, were used in these experiments because of their high reproductive capacity and fertility [16,18,19]. Transgenic PINK1 fish were created from this strain of fish. They were staged in hours post-fertilization (hpf) or days post-fertilization (dpf) based on [20]. The characterized antisense morpholino oligonucleotide (MO) from our previously published paper was used to inhibit PINK1 function [16]. The ethical permits and approval for the experiments were obtained from the Regional Government of Southern Finland, numbered ESAVI/3623/04.10.03/2012.

### Tol2 transgenic fish design

Transgenic fish were created by the Tol2 method using a gateway cloning kit (Invitrogen, Carlsbad, USA). The primers used for amplifying the gene promoter were as follows: GGGGACAAGTTTGTACAAAAAAGCAGGCTAATGATGCATCTCAGTCATTC as the forward primer and GGGGACCACTTTGTACAAGAAAGCTGGGTCTTTACTGACATTTTGAGCCAA as the reverse primer. The primers were designed to add attB sites to the final product to allow for insertion into the donor vector pDONR221 when performing the first reaction step of the gateway cloning. The clone was PCR verified and the insert was transformed into the destination vector pXIG-cfos-GW [21], and the sequence was then verified. The transposase mRNA was made from the pCS-TP vector as described earlier [22,23]. Both the transposase mRNA and the donor plasmid were injected into the zebrafish embryos at the one-cell stage at 25 ng/μl each. We outcrossed the founder fish (F0) with wild-type fish and collected the progeny embryos (F1). GFP expression was visualized in the injected embryos, and three founder fish lines differing in the levels of GFP expression were identified. One of these founder lines was chosen based on the maximum GFP expression pattern and named *Tg*(*pink1:EGFP*). These fish were crossed to produce stable line embryos (F2), which were grown until adults, and experiments were carried out on the F2 generation onwards. 

### Immunohistochemistry (IHC)

Embryos of different stages from 3 days post-fertilization (dpf) until 8 dpf were fixed with 4% PFA overnight at +4 °C. The samples were washed with PBST 3 x 30 min at RT followed by a pre-incubation with PBST, 1% DMSO, and 4% normal goat serum (NGS) for 4 hours to o/n at RT. The anti-GFP, chicken IgY fraction (Invitrogen, Carlsbad, USA) was used to stain the transgenic fish at 1:500 dilution o/n in PBST with 2% NGS at +4 °C. The other antibodies used were those against tyrosine hydroxylase (TH), PINK1 and 5-hydroxy tryptophan (5-HT) at 1:1000 dilution (Kaslin and Panula, 2001,Sallinen et al., 2010,Kaslin and Panula, 2001). The samples were handled and treated as has previously been described [16].

For brain sections, adult brain samples were dissected and fixed in 4% PFA overnight, followed by 20% sucrose for the following incubation overnight at +4 °C. Cryosections were made by embedding the brain samples in the embedding matrix, maintaining the section thickness at 25 µm for all the samples. 

Immunofluorescence samples were mounted with 80% glycerol and examined under a Leica TCS SP2 AOBS confocal microscope, using an argon laser (488 nm) and green diode laser (561 nm). The emission wavelength was set to 500–550 nm for Alexa 488 and 600–750 nm for Alexa 561, as described earlier [24]. Stacks of images were taken at 1.2-µm intervals, collected by sequential scanning. Frame averaging was carried out, and the maximum intensity projection algorithm was used to produce the final images with Leica Confocal Software. In each individual experiment, samples were scanned using the same parameters. Pictures were then compiled in Corel Draw 12 software (Corel Corporation, Ottawa, Canada). 3D volume rendering and the fluorescence intensity measurement was performed using the Imaris MeasurementPro module in Imaris 6.0 software (Bitplane AG, Zurich, Switzerland). Each of the IHC experiments included at least 12 individuals per group for imaging, and each set of experiments was repeated at least three times.

### In situ hybridization (ISH)

The larval fish at 3 dpf, 7 dpf and 8 dpf were fixed with 4% PFA o/n at +4 °C. The brains were dissected from 7- and 8-dpf-old larvae and stored in 100% methanol until further use. Digoxigenin (DIG)-labeled probes were made for *th1, th2, dat*, and *pink1* as described earlier [13]. The ISH protocol from Thisse et al. was used to perform the experiments [25]. Image analysis was conducted as described earlier [16,26,27].

### Chemicals and drug treatment

Hydrogen peroxide (H_2_O_2_) (Merck, NJ, USA) was used at a concentration of 5 µM for 20 min to create oxidative stress similar to the environment for the studies on wild-type and transgenic fish. L-Glutathione Reduced (LGR) (G4251) (Sigma-Aldrich, St. Louis, MO, USA) was used at a concentration of 100 μM for 24 h after H_2_O_2_ treatment. The dose and duration of LGR treatment had been tested and previously used on the morphants [13]. The samples were washed with E3 fish medium followed by the application of LGR for 24 hours. They were then processed for further experimentation. 

### Q-RT-PCR

RNA samples were isolated from pooled embryos from untreated and H_2_O_2_-treated wild-type fish (30 embryos/sample) with an RNAEasy minikit (Qiagen) at 3 dpf. RNA was reverse transcribed to produce cDNA using superscript reverse transcriptase-III (Invitrogen) primed with oligo (dT) primers according to the manufacturer’s instructions. Similar instrumentation and procedures for real-time PCR were followed as described earlier [13]. 

## Results

### Characterization of PINK1 transgene expression

**Figure 1:**
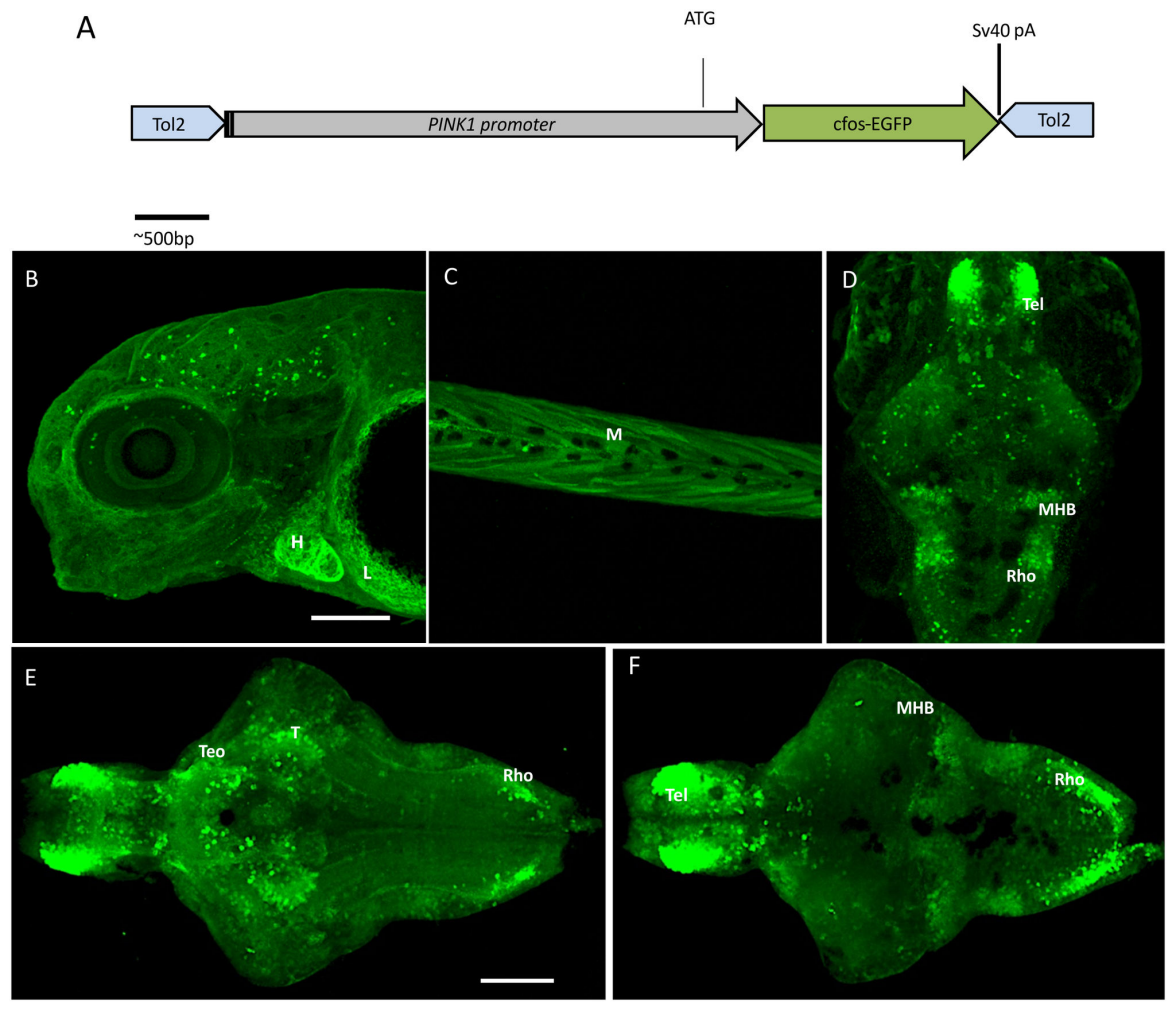
The PINK1 promoter construct and expression pattern in zebrafish larva of the *Tg*(pink1:EGFP) line. A. Construction of the PINK1 promoter DNA fragment comprising the sequence -2 kb upstream of the 5’ end of the *pink1* gene and including the ATG start site. This is inserted in the cloning site in the Tol2 transposon vector pCS-cfosGFP, which contains a small intronic sequence and a GFP expression cassette having minimal cfos activity [21]. B-F. Confocal z-stack images of the whole-mount *Tg*(pink1:EGFP) larval stages. B. GFP expression as observed in peripheral tissues at different stages of development by GFP IHC. In a lateral view of 4 dpf whole mount larvae, expression was observed in the heart (H) and liver (L); anterior to the left. C. Expression in the muscle of the 4-dpf larvae in a lateral view (M); anterior to the left. D. The whole mount GFP expression at 5 dpf; dorsal views, anterior to the top, showing pronounced immunoreactive cells in brain regions such as the telencephalon (Tel), mid-hindbrain boundary (MHB) and rhombencephalon (rho). E- F. Dorsal and ventral views of the brain of 7-dpf *Tg*(pink1:EGFP) fish; anterior to the right. E. In the ventral view, the strongest GFP expression is seen in the telencephalon (Tel), anterior part of optic tectum (TeO), thalamus (T), and the lateral rhombencephalon (rho). F. The dorsal view shows the prominent expression in the mid-hindbrain boundary (MHB) along with Tel and rho. Scale bar represents 100μm. GFP – Green fluorescent protein, PINK1 – PTEN-induced putative kinase 1.

**Figure 2:**
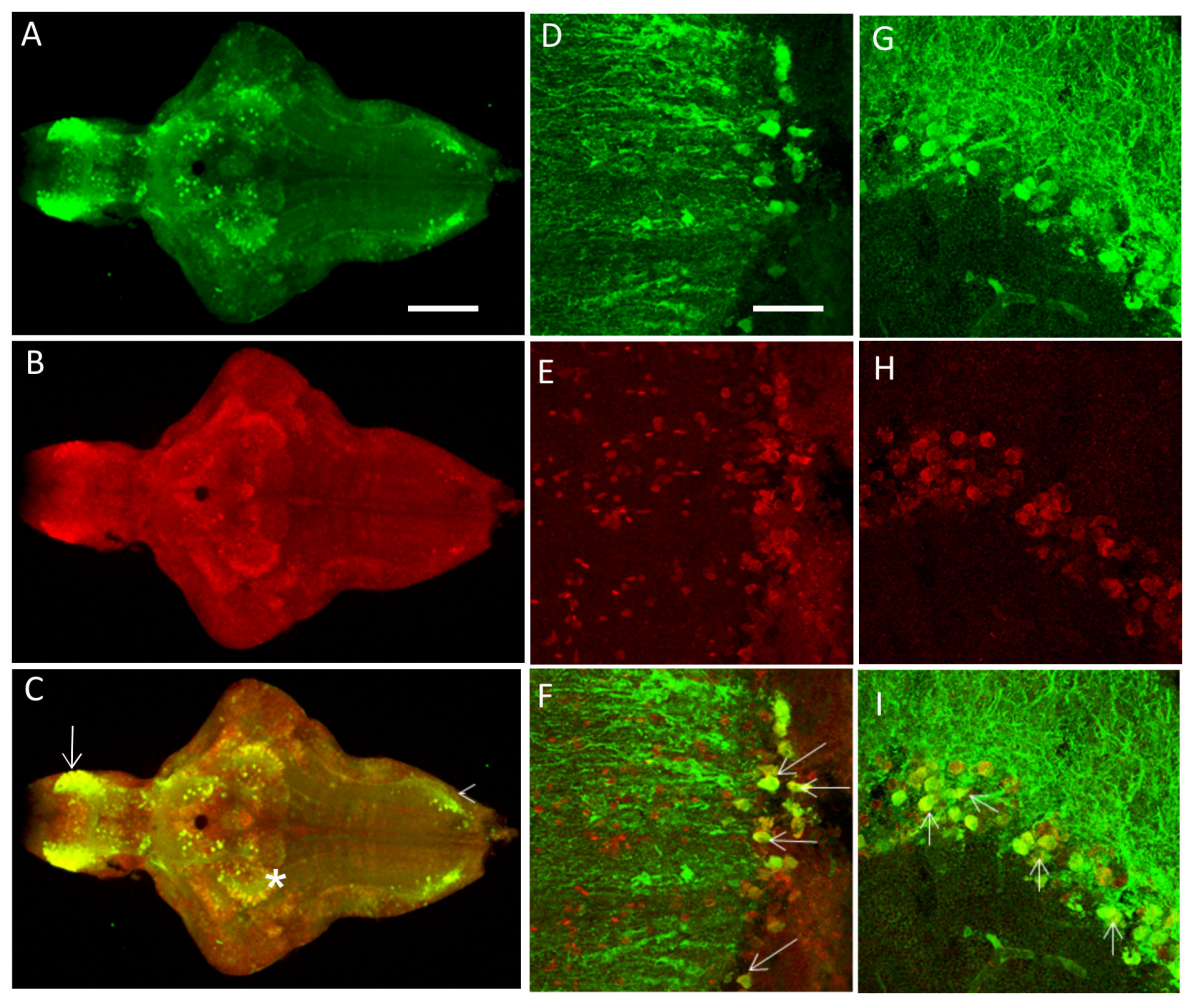
A-C. The PINK1-ir colocalizes with the GFP expression pattern in the brain of 7-dpf fish from the transgenic line in most parts of the brain. Colocalization was visualized in the telencephalon (arrow), the thalamic region (*), and the rhombencephalon (arrowhead). D-L. Representative higher magnification images from adult brain sections (25 µm) for PINK1-ir and GFP-ir. D-F represents the telencephalic region in the saggital plane. G-I represents the thalamic region, and J-L represents the rhombencephalic region from the coronal plane. GFP – Green fluorescent protein, PINK1 – PTEN-induced putative kinase 1, ir – Immunoreactivity.

The zebrafish PINK1 gene is located on chromosome 23 and shares 53% and 54% sequence identity with humans and mice, respectively, with a highly conserved sequence in the functional domains. The *pink1* transcripts can be detected as early as 3 hpf and expression persists throughout adulthood [16]. Several promoter constructs were produced and tested to identify at least the minimal promoter activity to drive PINK1 expression. A functional promoter construct included a 2-kilobase fragment of the 5'-flanking region from the translational start site and contained transcriptional regulatory sequences that were able to drive strong and ubiquitous GFP expression after injection into a single-cell embryo. This was created by the Tol2 transposition vector system [21] (Figure 1 A). In stable *Tg*(*pink1:EGFP*) transgenic lines, GFP was expressed in distinct regions from embryo to adulthood. The highest expressing regions were visualized in the brain, heart, liver and muscle by IHC on whole-mount fish embryos with GFP antibodies (Figure 1B-C). From the dorsal side of the intact brain, it was apparent that PINK1 GFP expression marked the mid-hindbrain boundary (MHB) (Figure 1D). Although low-level transgene expression showed a ubiquitous expression pattern, there were areas of higher expression in the brain. Analysis of the brain regions showed the highest GFP expression in the telencephalon (Tel), optic tectum (TeO), thalamus (T), and in discrete regions of the hypothalamus and rhombencephalon (rho) (Figure 1E-F). The GFP expression often colocalized with the PINK1-ir, suggesting that expression of the transgene follows the endogenous *pink1* expression pattern (Figure 2). The overlap was consistent with our previously published study on PINK1 [16]. The expression pattern in the brain at 7 dpf was compared with the pattern of TH immunoreactivity (ir) and 5-HT-ir (Figure 1). Although overlap in some of the cells was seen with TH and 5-HT immunoreactive regions, it was not apparent in all the cells (Figure S1 A–F). Transgene expression in the larval and adult brain sections was analyzed and a schematic representation of the highly apparent PINK1 transgene expression in zebrafish brain was prepared, as shown in Figure S2 A. 

### Oxidative stress

**Figure 3:**
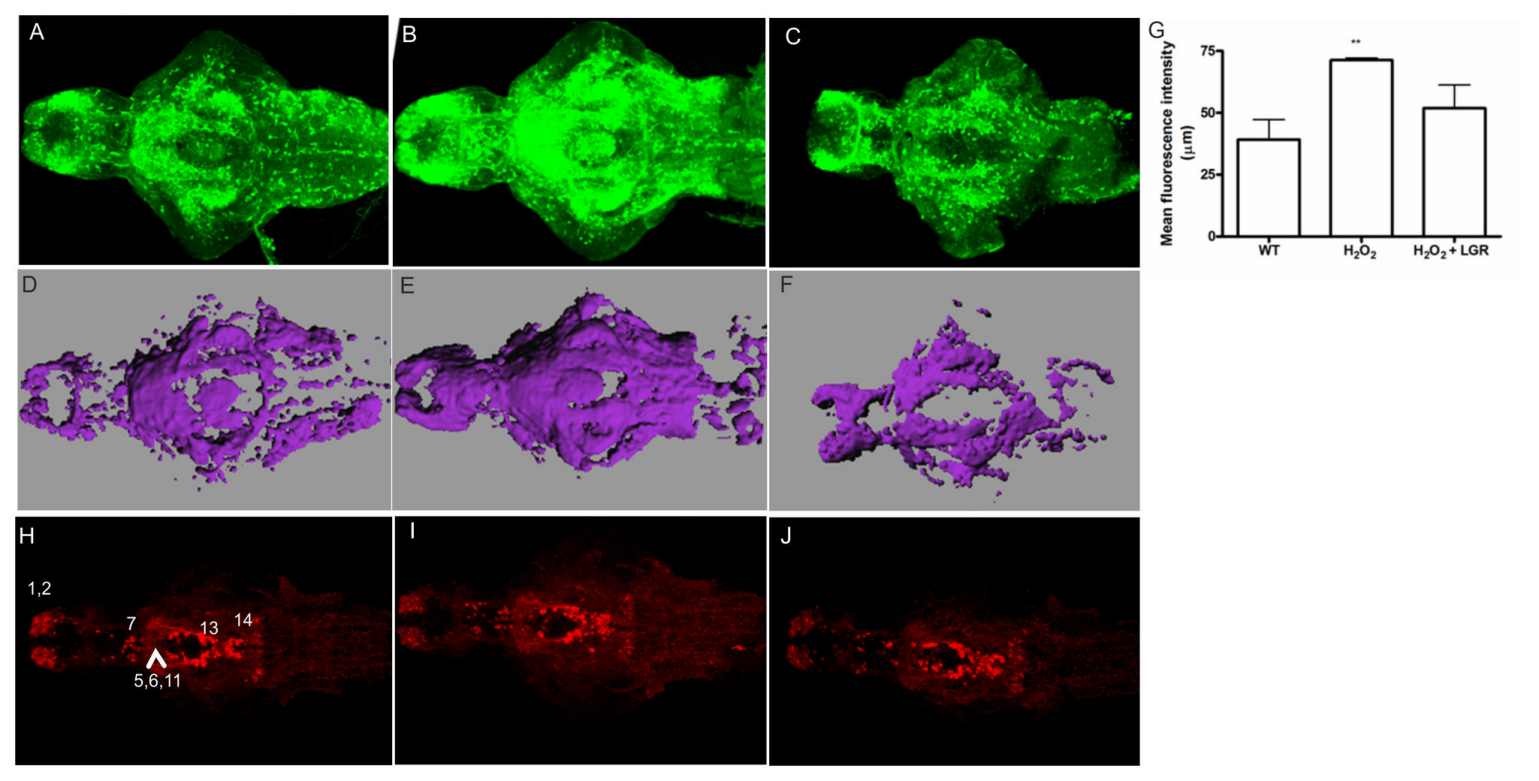
The effect of H_2_O_2_ on GFP and TH1 expression in *Tg*(*pink1:EGFP*) larval fish. A-C. GFP-ir at 7 dpf in the zebrafish larvae. GFP-ir increases in the H_2_O_2_-treated fish (B) as compared to the control fish (A). The effect is rescued in the LGR treated fish (C). D. Mean fluorescence intensity values of the WT, H_2_O_2_-treated group and the H_2_O_2_+LGR treated group. A significant change in the H_2_O_2_-treated group was observed (p < 0.01). E-G. A three-dimensional image to perform volume rendering was constructed using the Imaris MeasurementPro module in Imaris software for the above-mentioned fish at a similar threshold intensity. The changes indicated as the changed total volume of the representative images for control (E), H_2_O_2_ (F) and LGR-treated fish (G). Scale bar represents 80 μm. H-J. TH-ir is unchanged among the control (H), H_2_O_2_-treated (I) and H_2_O_2_ together with LGR (J) groups of larval fish. No change in the pretectal (7) and preoptic, thalamic or pre-tectal nucleus (5,6,11) cell populations was seen. Other visible groups in the olfactory bulb and ventral telencephalic nuclei (1,2), periventricular hypothalamus and posterior tuberculum (13), and the locus coeruleus (14) also displayed no change in TH-ir. Scale bar represents 100 μm. A-C and H-J. H2O2 – Hydrogen Peroxide, GFP – Green fluorescent protein, ir – immunoreactivity, LGR – L-glutathione reduced, PINK1 – PTEN-induced putative kinase 1, TH – tyrosine hydroxylase.

To investigate the role of stress in PINK1 regulation, we used the transgenic line as well as wild-type larvae. The transgenic larvae and the wild-type larvae were subjected to a mild heat shock or treated with 5 μM H_2_O_2_. Changes in transgene expression were observed by performing IHC with the GFP antibody, and in wild-type fish, gene expression was detected by ISH and transcript levels were measured by q-RT-PCR. To determine whether *Tg*(*pink1:EGFP*) reflects *pink1* responses to oxidative stress, we subjected the transgenic larvae to a very low concentration of H_2_O_2_ for 20 min and fixed the larvae to perform IHC with GFP antibodies. The PINK1 transgenic larvae showed an increase in GFP-expressing cells in the whole brain compared to the control fish without treatment (Figure 3 A–C). This effect was reduced in the presence of the drug L-Glutathione Reduced (LGR). The change in expression levels of the transgene was pronounced. We therefore created a 3D image using the ImarisMeasurementPro module in Imaris software (Bitplane) and visualized the volume of cells expressing the transgene in different planes. The apparent increase in transgene expression in certain regions of the brain could be easily visualized by the 3D representation. The volume of GFP-expressing cells in the H_2_O_2_-treated group was considerably larger than that of control brain and LGR-treated brain samples from the *Tg*(*pink1:EGFP*) line (Figure 3 D–F). Increased expression was evident in the telencephalon and midbrain, extending to the hindbrain. The mean fluorescence intensities of the groups were measured and mapped in a graphical manner, as shown in Figure 3 G. The *Tg*(*pink1:EGFP*) fish were subjected to heat shock, but no effect on the expression levels of the transgene was observed (data not shown). We then analyzed the effect of H_2_O_2_ treatment on TH-ir in dopaminergic neuron groups and found no differences between the control and H_2_O_2_-treated fish (Figure 3 H-I). However, the monoclonal antibody used only identifies the TH1 protein and not the TH2 protein [26]. No significant differences were detected in dat (dopamine transporter) expression as observed by q-RT-PCR and ISH (Figure S3).

**Figure 4:**
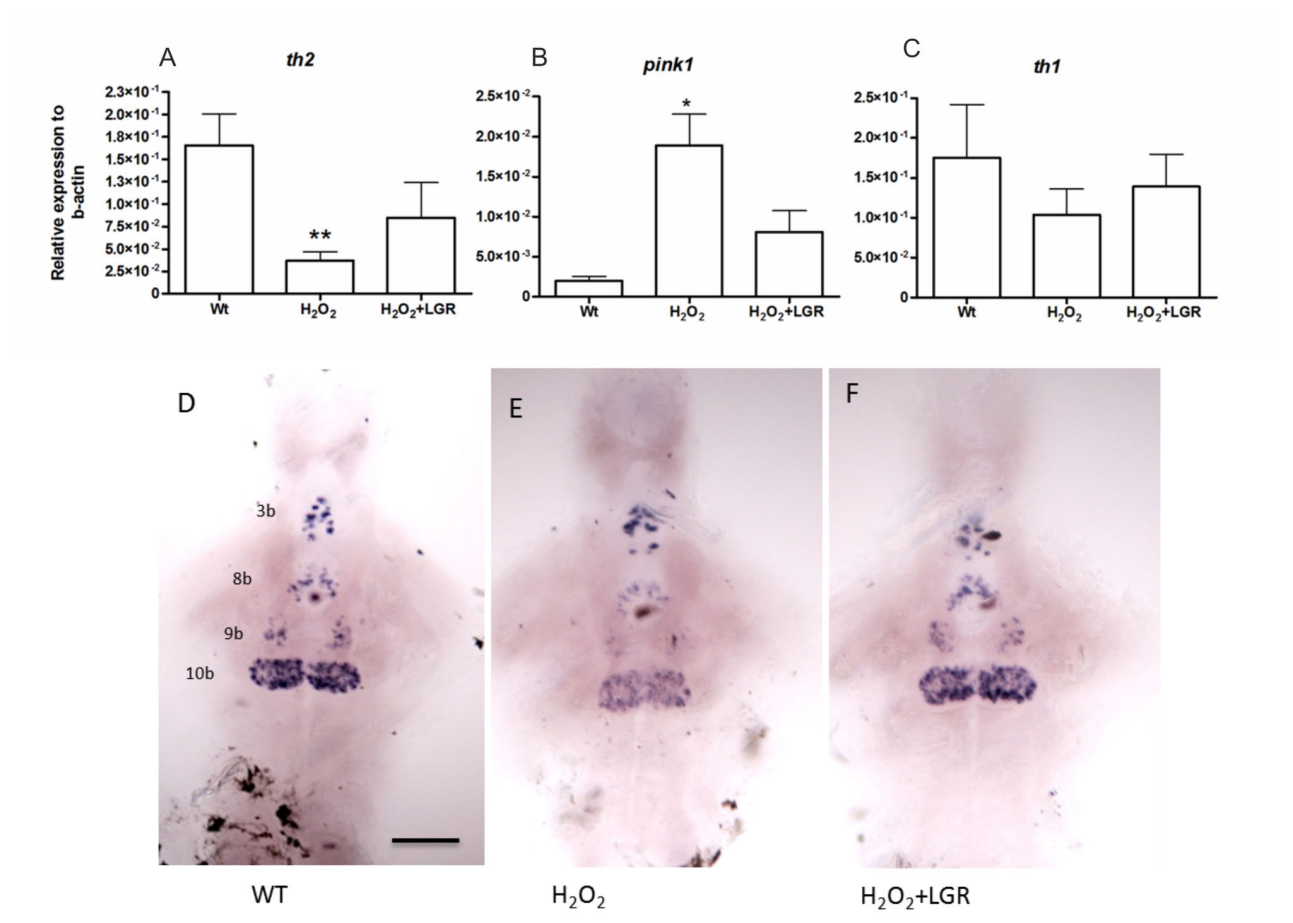
In-situ hybridization and q-RT-PCR at 3dpf in wild type fish following treatment with H_2_O_2_ and H_2_O_2_+LGR. A. The *th2* transcript levels significantly decreased in the H_2_O_2_ treated samples, verified by q-RT-PCR. B. The *pink1* transcript levels increased significantly in the H_2_O_2_ treated as compared to wild type samples. C. No significant change in the *th1* transcript levels was detected between the groups. Only a declining trend was observed. Unpaired t-test (* p-value < 0.05, ** p-value< 0.01). D-F. The expression of *th2* mRNA is reduced in group 10b of the 7-dpf H_2_O_2_-treated fish as compared with untreated control fish. In the LGR-treated groups at 8 dpf, the decrease in *th2* mRNA expression was prevented. The *th2*-expressing neuron groups 3b, 8b, 9b, 10b are identified according to [26]. Scale bar represents 100 μM. *th1* – tyrosine hydroxylase1, *th2* – tyrosine hydroxylase 2, *pink1* – PTEN-induced putative kinase 1.

To examine whether H_2_O_2_ treatment affected *th2* expression, we studied the transcript levels of *th2, pink1*, and *th1* by q-RT-PCR in wild-type larvae at 3 dpf (Figure 4 A–C). A decrease in *th2* and an increase in *pink1* transcripts were detected, while no change occurred in *th1* levels. These effects were also rescued by LGR administration, as revealed by q-RT-PCR. The Turku strain wild-type larvae were subjected to H_2_O_2_ at 7 dpf, and the effect of LGR on *th2* and *pink1* expression was studied at 8 dpf. *In situ* hybridization was performed at 7 dpf, because changes in *th2* levels could be monitored effectively in all the individual cell groups expressing *th2*. In agreement with the q-RT-PCR results, the signal intensities of *th2* were reduced in the H_2_O_2_-treated groups, and were rescued by LGR (Figure 4 D–F). 

### Effect of the drug LGR on pink1 morphants

The loss of dopaminergic neurons is a characteristic feature of PD. In zebrafish, the dopaminergic system has been visualized by TH immunoreactivity. Following the identification of the second *th* gene, we have focused on the differential expression of *th1* and *th2* in the current study. Our previous study on *pink1* revealed that LGR was an effective drug that could normalize erythropoiesis in *pink1*-deficient fish [13]. Here, we utilized the same drug regime to treat the *pink1* morphants and to study the effects on *th1* and *th2* expression, which was decreased in the morphants, as reported earlier at 3 dpf [16]. 

**Figure 5:**
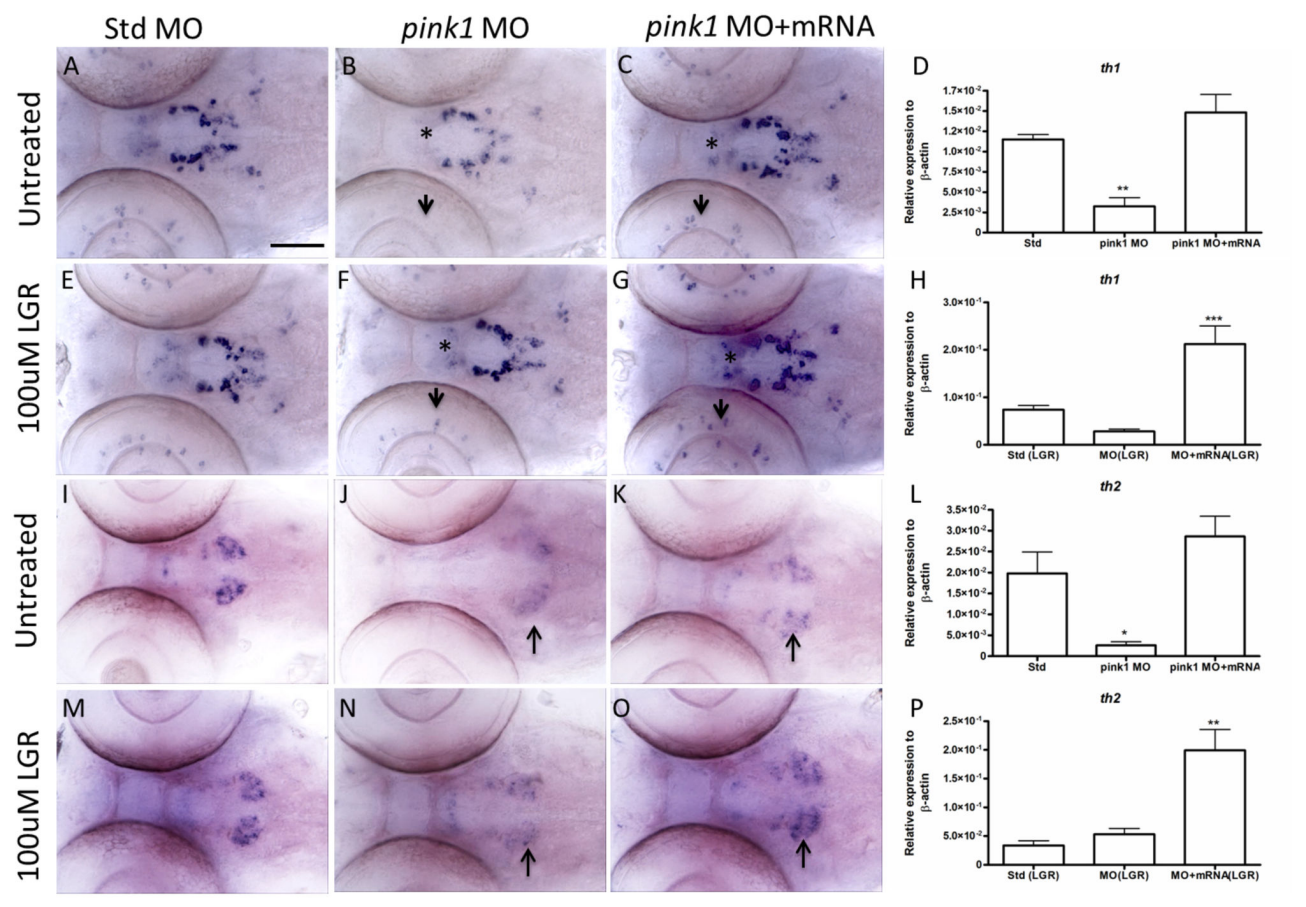
Expression levels of *th1* and *th2* mRNA at 3 dpf in *pink1* morphants untreated and treated with LGR. A-C. Altered *th1* expression in the untreated std ctrl MO, *pink1*MO, and *pink1*MO+mRNA groups. A decrease in *th1* expression in the population 5,6,11 (*) in morphants (B) was observed as compared to the ctrl MO group (A). The expression could be rescued by *pink1* mRNA co-injection (C). D. Representative quantitative graph for the untreated group. E-G. The expression of *th1* ISH in LGR-treated groups. The drug LGR rescued the reduced *th1* cells in the eyes and also group 5,6,11 of the morphants (E) as compared to the ctrl MO group (D). The expression is enhanced in the rescue group with the combined effect of LGR and *pink1* mRNA (F). H. Graphical representation of the transcript levels for *th1* in the LGR-treated group. A significant change in the *pink1* mRNA co-injected group. I-K. The expression of *th2* ISH in the untreated std MO, *pink1*MO, and *pink1*MO+mRNA groups. A loss of *th2* was observed in group 10b in the *pink1* morphants (H) as compared to ctrl MO (G). The *pink1* mRNA rescued *th2* expression in group 10b (I). L. The representative *th2* transcript levels determined by q-RT-PCR for the untreated group. M-O. The expression of *th2* ISH in LGR treated groups. LGR-treated *pink1* MO shows a slight increase in the *th2* 10b group (K) as compared to ctrl MO (J). The *pink1* mRNA along with LGR completely rescues the loss of *th2* in the 10b cell group (L). P. Representative q-RT-PCR results for the *th2* LGR-treated group. A significant change in the *pink1* mRNA co-injected group together with LGR significantly increased the *th2* transcript levels. Figures D and L present confirmation of *th1* and *th2* transcripts downregulation in the *pink1* morphants. Figures H and P present the additive rescue effect of LGR together with *pink1* mRNA on the morphants. The Bonferroni Hochberg multiple comparison statistical test was applied for the q-RT-PCR results (* p < 0.05, ** p < 0.01, *** p < 0.001). ISH – in-situ hybridization, LGR – L-glutathione reduced, MO – morpholino oligonucleotides, *pink1* – PTEN-induced putative kinase 1, q-RT-PCR – quantitative real-time PCR, *th1* – tyrosine hydroxylase1, *th2* – tyrosine hydroxylase 2. Scale bar represents 100 μm.

In our previous study, using the same *pink1* MO, we demonstrated that *th1* expression declined in the morphants in the cell group complex of 5,6,11, which represents the preoptic, thalamic and pre-tectal nucleus in the diencephalon [28]. This cluster of neurons has also been named as the ventral diencephalic group (vDC) [29]. In the current study, we subjected the wild-type fish to *pink1* MO, standard MO, and co-injected a group with *pink1* MO and *pink1* mRNA, and examined the effect of LGR on the expression of *th1* and *th2* (Figure 5). The loss of group 5,6,11, representing *th1* neurons in *pink1* morphants, was rescued by *pink1* mRNA, as has been observed by ISH (Figure 5 A–C). The effect was verified by q-RT-PCR (Figure 5 D). The effect of LGR on morphants slightly increased *th1* expression in the *pink1* morphants (Figure 5 E–G). LGR treatment could also rescue the *th1* amacrine cells, which lost visible expression, in the retina of the morphants and partially in the pre-tectal region (Figure 5 B and F). In the rescue group (Figure 5 F), the additive effect of *pink1* mRNA and LGR enhanced *th1* expression to a larger magnitude than that seen in the standard MO group, as was quantified by q-RT-PCR (Figures 5 H). 

We analyzed *th2* expression patterns in the standard MO, *pink1* MO and *pink1* MO + *pink1* mRNA co-injected samples without treatment (Figure 5 I–K) and with LGR treatment (Figure 5 M–O). LGR improved *th2* expression in the *pink1* morphants in the dopaminergic cell group 10b (as defined in [26]), and the effect of the drug with *pink1* mRNA was stronger than that of *pink1* mRNA alone (Figures 5 K, O). The loss of *th2* expression has been verified in the morphants by q-RT-PCR (Figure 5 L). The drug LGR was quite effective for *th2* cell group enrichment, as can also be seen from the q-RT-PCR results (Figure 5 P).

## Discussion

The goal of this study was to develop and validate a transgenic model that will help in the interpretation of the precise function of the PINK1 *in vivo*, and to study the effects of oxidative stress and PINK1 dysfunction on *th* genes. Our data objectively demonstrate that oxidative stress significantly downregulates *th2* in distinct neuronal cell populations and upregulates *pink1* in larval zebrafish. Both effects were normalized by treatment with L-Glutathione Reduced. Reduced glutathione could also rescue, along with *pink1* mRNA, both *th1* and *th2* expressing cell populations, which were lost in *pink1* morphants.

The existing experimental evidence strongly suggests that PINK1 protects neurons against oxidative stress and also neurotoxins. In neuroblastoma cells treated with toxins such as staurosporine and MG132, PINK1 overexpression suppresses toxin-induced damage [30,31]. The development of the transgenic line for PINK1 provided us with a tool to visualize and characterize the dynamic changes in its expression in live zebrafish. The *Tg*(*pink1:EGFP*) line provided strong transgene expression that was integrated into the germline and sustained over several generations. Even though PINK1 is ubiquitously expressed, transgene expression was apparently higher in some regions than others. It has not been ruled out that the patterns we see could be attributed to the lack of some regulatory elements in the 2 kilobase genomic segment used in this study. This needs to be taken into account in future use of this fish line. This phenomenon with ubiquitous promoters for genes such as *ubi* and β-actin has previously shown that these promoters, with strong expression during development, gradually become silenced and restricted to certain cell groups, and are not expressed in others [32,33]. This is either due to random integration into the genome or due to special cell type-specific functions. Analysis of the detailed expression pattern of PINK1 in zebrafish has been lacking. In the mouse and rat, *PINK1* mRNA is expressed unevenly in neurons of the cortex, striatum, thalamus, brainstem, and cerebellum [34]. In humans, *PINK1* expression is highest in the hippocampus, substantia nigra and cerebellar Purkinje cells [35]. We have reported the expression in different brain regions with PINK1 antibody [16], and in the transgenic fish we were able to visualize the expression pattern throughout their development and confirm the ubiquitous expression pattern in the vast majority of cell types by GFP IHC. Apparently higher expression was observed in the telencephalon, anterior part of optic tectum, thalamus, rhombencephalon, and mid-hindbrain boundary in the larval and adult brain sections. The expression pattern of the transgene was very similar to the *pink1* fish pattern, which indicated that the transgenic fish recapitulate endogenous gene expression. The *pink1* promoter is not expressed at same level in all cell types. These effects are anticipated for these randomly integrated transgene constructs [32].

### Effect of H_2_O_2_ on the transgenic fish line

Based on previous studies in different cell lines, PINK1 has neuroprotective activity, most likely by antagonizing mitochondrial dysfunction caused by oxidative stress and neurotoxins [30]. PINK1 overexpression in the neuroblastoma cell line SHSY5Y could rescue cell death caused by neurotoxins [31]. Postmortem investigations of PD patients and findings from studies using the selective nigral toxin MPTP have revealed increased iron levels, decreased levels of reduced glutathione (GSH), and impaired mitochondrial function [15], which supports the hypothesis that oxidative stress contributes to nigral cell degeneration in PD. Administration of H_2_O_2_ leads to similar damage in different strains of zebrafish [36]. The free radical generator H_2_O_2_ has been used in the oxidative stress model of PD in the SHSY5Y cell line as an *in vitro* model [37]. Pink1 overexpression protects cells against oxidative stress, which is achieved by TRAP1 phosphorylation [38]. The H_2_O_2_ treatment concentration in this study was selected based on previous studies, which have reported that doses below 150 μM are safe for zebrafish embryos and cause no abnormalities [39]. Therefore, we decided to use a very low concentration (5 μM) of H_2_O_2_ for treatment that would have no deleterious effects on the normal fish phenotype or behavior, but could possibly still trigger a physiological stress response. This concentration was indeed sufficient to alter the expression pattern of *pink1* in the transgenic line, and *pink1* and *th2* in the wild-type fish, but it was not enough to significantly disrupt the transcript levels of *th1*. In summary, *th2* together with *pink1* was more sensitive to H_2_O_2_ than *th1*. 

### Differential regulation of the catecholaminergic systems

We could see that TH-ir, which mostly detects *th1* [26], did not change considerably in H_2_O_2_-treated samples, while *th2* mRNA levels declined. This result was also confirmed by q-RT-PCR, in which the *th2* gene was significantly downregulated, while the *th1* transcript did not show any significant changes. The two *th* genes have been known to show differential regulation in teleosts [40]. Additional studies have been performed using different markers for the dopaminergic system, which have revealed that *th2* neurons are dopamine-synthesizing cells, in addition to *th1* cells [29,41]. Dopamine cells are very vulnerable to slight changes in oxygen, because TH is the rate-limiting enzyme in catecholamine synthesis and requires oxygen as its cofactor. Therefore, low levels of cellular oxygen or environmental oxygen would affect the levels of both *th* genes. In zebrafish, *th2* is known to be more vulnerable to slight changes in oxygen than *th1* [42]. In this study, the applied concentration of H_2_O_2_ was quite low and did not significantly affect the *th1* levels, but was sufficient to alter *th2* levels. A recently published paper stated that *th2* had tryptophan hydroxylase-like activity [43]. Independent verification has not been published and this question was not addressed in this study. The mechanisms of differential regulation of the two non-allelic *th* forms need further investigation. A mutant for *th2* could be beneficial in order to understand the precise function of *th2* in the brain.

In *pink1* morphant larvae, the *pink1* mRNA could rescue the detrimental effects caused by the knockdown. When LGR was added, in addition to the *pink1* mRNA, the rescue effect was clearly enriched. LGR is a thiol antioxidant and protects cells from various cytotoxic insults, as indicated in a study conducted on rabbits [14]. Glutathione is administered in the reduced form, as it can more easily cross the cell membrane than glutathione itself [44]. It has also been shown that the level of glutathione within the cell is quite critical for the cellular redox balance [14]. This provides suitable evidence that PINK1 and glutathione might operate on the same pathways. The present study contributes to our understanding of the mechanism of PD pathogenesis in the oxidative stress-mediated pathway. 

## Conclusions

This study suggests that PINK1 activity could potentially serve as a valid target for neuroprotection in PD. The drug L-Glutathione Reduced could effectively normalize the decline in *th2* and *th1* expression in the *pink1* morphants and it could also recover the altered levels of *th2* and *pink1* in the groups subjected to H_2_O_2_ in the oxidative stress model. This protective effect of glutathione on the dopaminergic system may prove significant for drug development targeted at the early phases of degenerative diseases such as PD. The zebrafish is now emerging as an alternative for modeling human disease and drug discovery [45,46]. The development of the *Tg*(*pink1:EGFP*) zebrafish has provided a useful tool for drug screening and the mechanistic analysis of oxidative damage in the central nervous system as a whole, in addition to analysis of the dopaminergic system and PD mechanisms. The *Tg*(*pink1:EGFP*) line potentially responds to subtle changes in the external environment and this makes it a valuable tool for study of environmental stressors on *pink1* gene function.

## Supporting Information

Figure S1 **Comparison of the GFP expression pattern with TH-ir and 5-HT-ir in Tg(pink1:EGFP) fish at 7 dpf.** D-F. TH ir compared to GFP distribution in the Tg(pink1:EGFP) fish. GFP is more widely distributed than TH-ir. A ventral view of the maximum projection images for TH and GFP. The cell populations and numbering are as in [28].
G-I. The 5-HT-ir is colocalized with GFP expression in the posterior recess of the paraventricular organ (arrowhead).
5-HT – 5-hydroxy tryptophan, TH – tyrosine hydroxylase, ir – immunoreactivity, GFP – Green fluorescent protein, PVOa – paraventricular organ anterior part, PVOi – paraventricular organ intermediate part, PVOp – paraventricular organ posterior part.
Scale bar represents 100 μm.
(TIF)

Figure S2 A. Schematic lateral view of pink1:egfp expressing regions in the zebrafish brain compiled from immunohistochemistry of larval and adult brain sections. The regions are marked as: Tel – telencephalon, Teo – anterior region of the optic tectum, Hy – hypothalamus, rho – rhombencephalon.
B-E. Cryosections of different regions of the adult zebrafish brain with immunoreactivity detected for TH (red), PINK1 (blue) and pink1:egfp (green). The cell populations of TH-ir are reported with numbers, and additional regions of GFP are marked with dotted lines.
Di – lateral zone of the dorsal telencephalon, ENd – endopeduncular nucleus, VOT – ventrolateral optic tract, TeO – tectum opticum, PTN – posterior tuberal nucleus, Hc – caudal zone of the periventricular hypothalamus, LCa – lobus caudalis cerebelli, ALLN – anterior lateral line nerves.
Scale bar represents 100 μm.
(TIF)

Figure S3 **The transcript levels of *dat* in WT and H_2_O_2_-treated fish.** A. Larval groups measured by q-RT-PCR. No significant level of transcript alteration was visualized amongst the groups.
B. No change in expression levels between the two groups was also observed by ISH.
Scale bar represents 100 µm.
(TIF)
