# Supplementary figures and images for: Oxidative Stress and Regulation of Pink1 in Zebrafish (Danio rerio)
Source: PLoS One. 2013 Nov 26;8(11):e81851. doi: 10.1371/journal.pone.0081851 (PMC3850071; doi:10.1371/journal.pone.0081851)

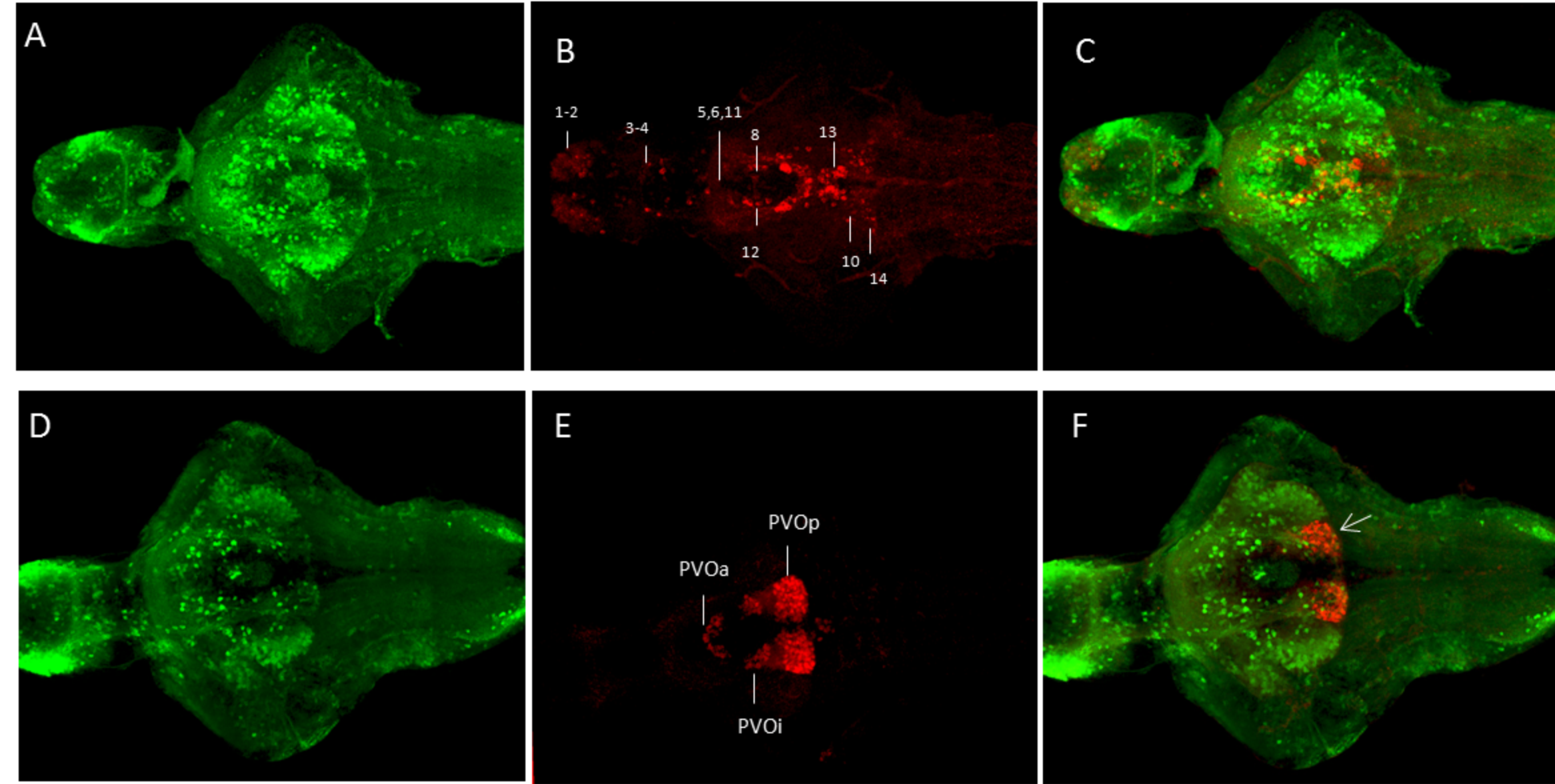

Supplement: Figure S1 — Comparison of the GFP expression pattern with TH-ir and 5-HT-ir in Tg(pink1:EGFP) fish at 7 dpf. D-F. TH ir compared to GFP distribution in the Tg(pink1:EGFP) fish. GFP is more widely distributed than TH-ir. A ventral view of the maximum projection images for TH and GFP. The cell populations and numbering are as in [28]. G-I. The 5-HT-ir is colocalized with GFP expression in the posterior recess of the paraventricular organ (arrowhead). 5-HT – 5-hydroxy tryptophan, TH – tyrosine hydroxylase, ir – immunoreactivity, GFP – Green fluorescent protein, PVOa – paraventricular organ anterior part, PVOi – paraventricular organ intermediate part, PVOp – paraventricular organ posterior part. Scale bar represents 100 μm. (TIF) [file pone.0081851.s001.tif]

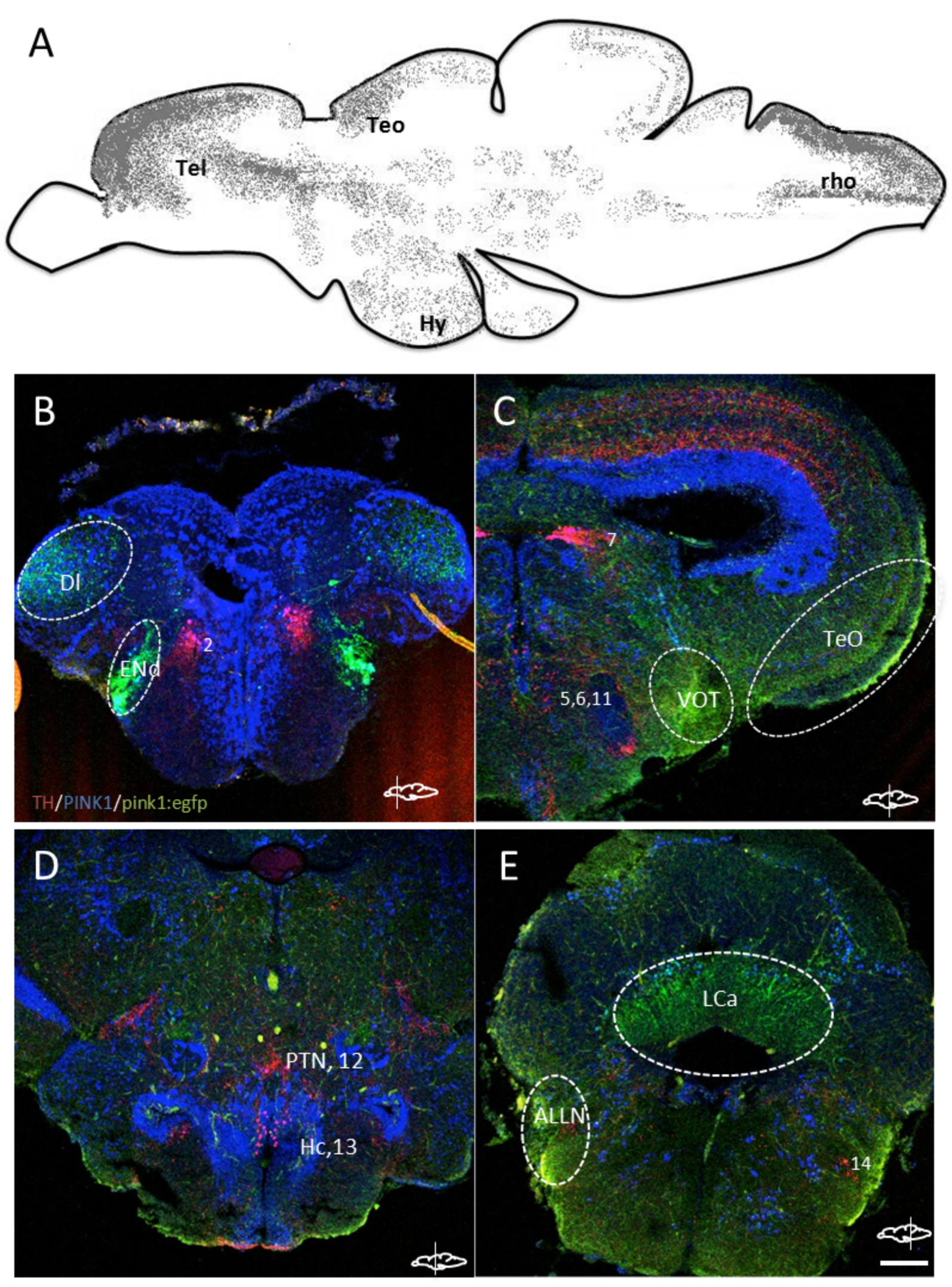

Supplement: Figure S2 — A. Schematic lateral view of pink1:egfp expressing regions in the zebrafish brain compiled from immunohistochemistry of larval and adult brain sections. The regions are marked as: Tel – telencephalon, Teo – anterior region of the optic tectum, Hy – hypothalamus, rho – rhombencephalon. B-E. Cryosections of different regions of the adult zebrafish brain with immunoreactivity detected for TH (red), PINK1 (blue) and pink1:egfp (green). The cell populations of TH-ir are reported with numbers, and additional regions of GFP are marked with dotted lines. Di – lateral zone of the dorsal telencephalon, ENd – endopeduncular nucleus, VOT – ventrolateral optic tract, TeO – tectum opticum, PTN – posterior tuberal nucleus, Hc – caudal zone of the periventricular hypothalamus, LCa – lobus caudalis cerebelli, ALLN – anterior lateral line nerves. Scale bar represents 100 μm. (TIF) [file pone.0081851.s002.tif]

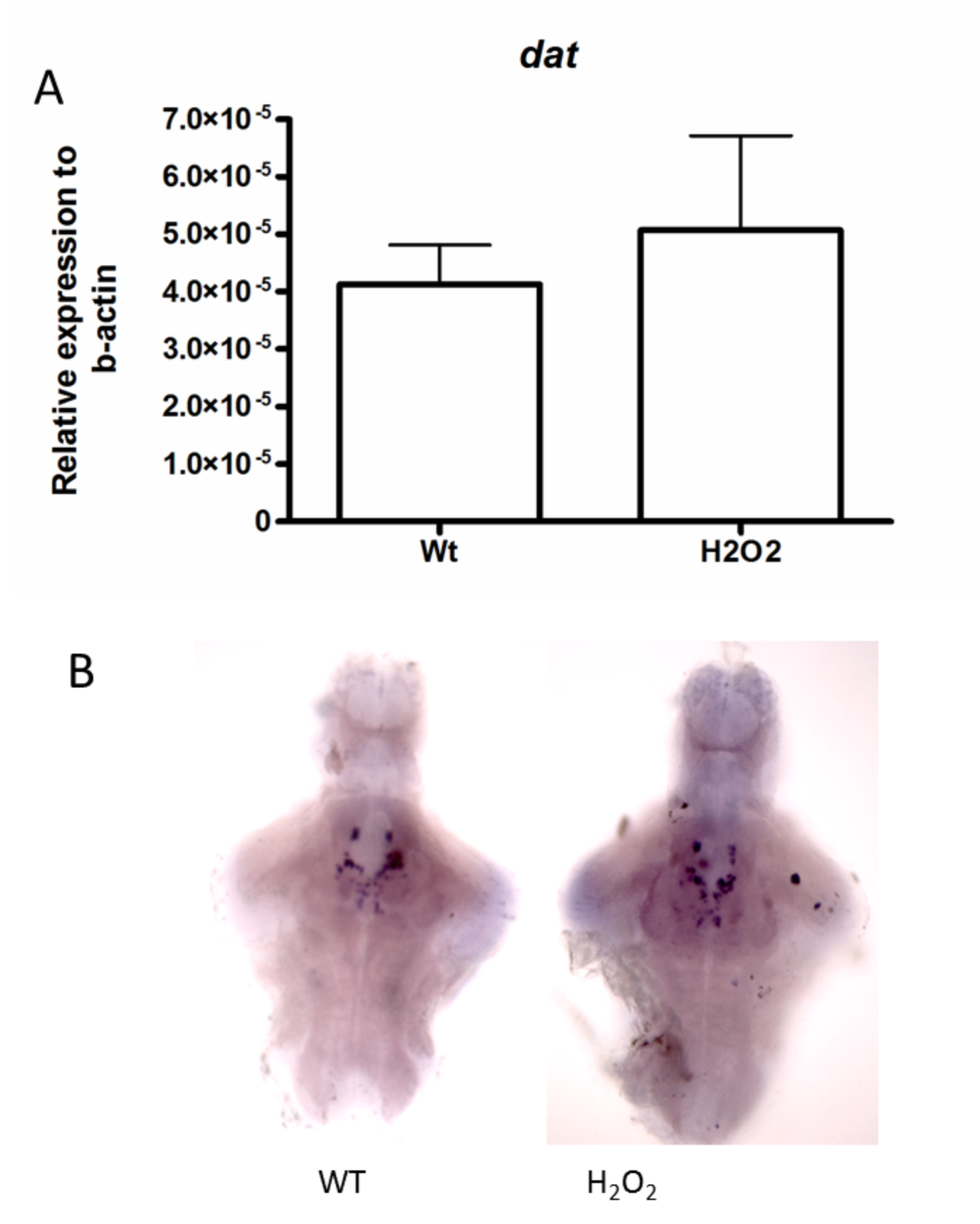

Supplement: Figure S3 — The transcript levels of dat in WT and H2O2-treated fish. A. Larval groups measured by q-RT-PCR. No significant level of transcript alteration was visualized amongst the groups. B. No change in expression levels between the two groups was also observed by ISH. Scale bar represents 100 µm. (TIF) [file pone.0081851.s003.tif]
